# Photoaging Mobile Apps in School-Based Tobacco Prevention: The Mirroring Approach

**DOI:** 10.2196/jmir.6016

**Published:** 2016-06-28

**Authors:** Titus Josef Brinker, Werner Seeger, Fabian Buslaff

**Affiliations:** ^1^ Universities of Giessen and Marburg Lung Center Member of the German Center for Lung Research Justus-Liebig-University of Giessen Giessen Germany

**Keywords:** tobacco, smoking, adolescents, photoaging, apps, secondary schools, adolescent smoking, tobacco prevention, smoking prevention, smoking cessation

## Abstract

**Background:**

Most smokers start smoking during their early adolescence, often with the idea that smoking is glamorous. Adolescent smoking can best be prevented through health education at schools. Interventions that take advantage of the broad availability of mobile phones as well as adolescents’ interest in their appearance may be a novel way to improve prevention.

**Objective:**

In this first pilot study, we aimed to use mobile phone technology in accordance with the theory of planned behavior to improve school-based tobacco prevention.

**Methods:**

We used a free photoaging mobile phone app (“Smokerface”) in three German secondary schools via a novel method called mirroring. The students’ altered three-dimensional selfies on mobile phones or tablets were “mirrored” via a projector in front of their whole grade. Using an anonymous questionnaire, we then measured on a 5-point Likert scale the perceptions of the intervention among 125 students of both genders (average age 12.75 years).

**Results:**

A majority of the students perceived the intervention as fun (77/125, 61.6%), claimed that the intervention motivated them not to smoke (79/125, 63.2%), and stated that they learned new benefits of non-smoking (81/125, 64.8%). Only a minority of students disagreed or fully disagreed that they learned new benefits of non-smoking (16/125, 12.8%) or that they were themselves motivated not to smoke (18/125, 14.4%).

**Conclusions:**

We have presented a novel method to integrate photoaging in school-based tobacco prevention that affects student peer groups and considers the predictors of smoking in accordance with the theory of planned behavior.

## Introduction

Most smokers start smoking during their early adolescence, often with a vision in their head of the kind of smoker they want to be [1]. In a recent paper, we introduced photoaging mobile apps that alter a person’s self-portrait (ie, a selfie) to predict future appearance [2]. These are considered a novel opportunity for smoking prevention after their effectiveness was first demonstrated by Burford et al [3]. In addition to this, many dermatology publications have called for a novel public health approach in light of new findings on the photoaging effects of smoking [4-8]. A photoaging approach is relevant for teenagers as evidenced by numerous publications demonstrating the influence of attractiveness on self-confidence and quality of life of adolescents [9-11].

According to a recent Cochrane analysis, adolescent smoking can most effectively be prevented by health educators in schools, but no data for photoaging interventions or their implementation in the school setting are available to date [4].

## Methods

To integrate these novel interventions into the school-based setting, we developed and tested the mirroring approach in a pilot study. Mirroring means that the students’ altered three-dimensional selfies on mobile phones or tablets were “mirrored” via a projector in front of the whole grade. We included a total sample of 125 Grade 7 students in our cross-sectional pilot study with an average age of 12.75 years (49/125, 39.2% female; 76/125, 60.8% male) attending three secondary schools in Germany. The mirroring approach was implemented by medical students from the Education Against Tobacco non-profit organization [12]. To increase familiarity with the photoaging app (called “Smokerface”) and students’ participation in the mirroring intervention, students were asked to download the app before our visit, via a letter 3 days in advance. By this means, 52% (21/41) of the grammar school students and 44% (16/36) of the general school students had the photoaging app on their mobile phone when we visited the schools.

**Figure 1:**
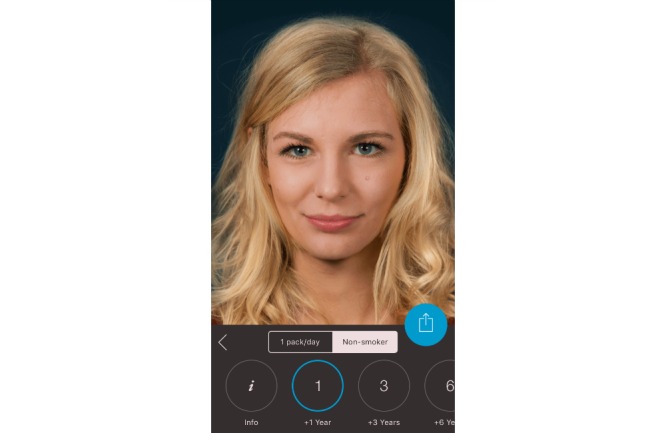
Screenshot from the Smokerface app illustrating the effects of non-smoking for 1 year.

**Figure 2:**
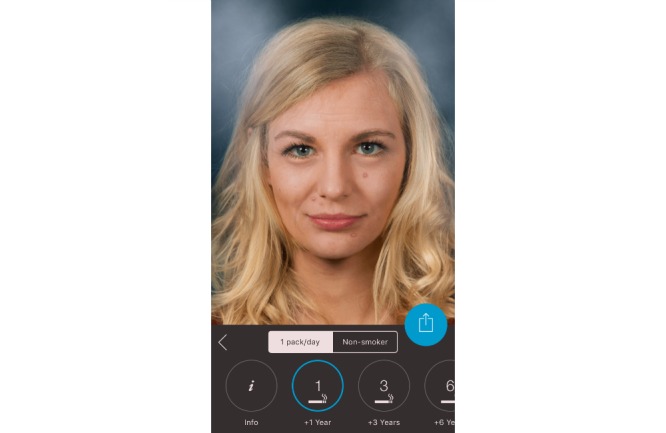
Screenshot from the Smokerface app illustrating the consequences of smoking a pack a day for 1 year.

**Figure 3:**
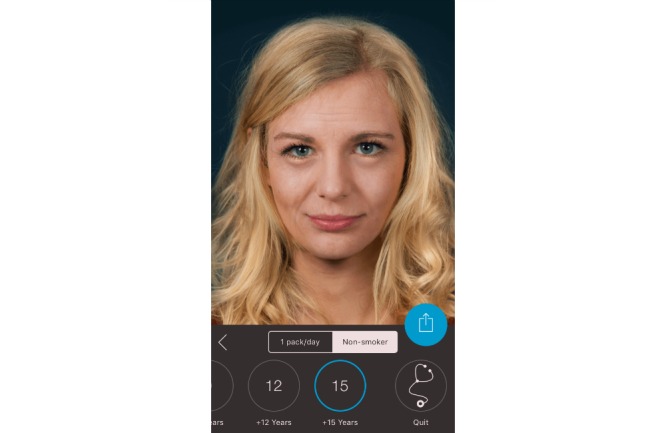
Screenshot from the Smokerface app illustrating the effects of non-smoking for 15 years.

**Figure 4:**
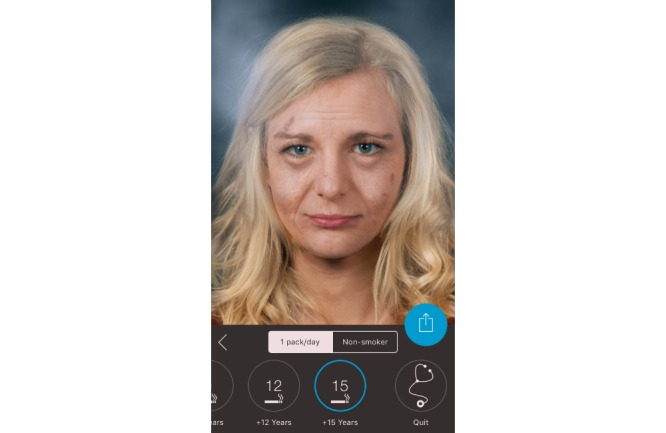
Screenshot from the Smokerface app illustrating the consequences of smoking one pack a day for 15 years.

In the first 10-minute phase, the displayed face of one student volunteer was used to show the app’s altering features to the peer group, providing an incentive for the rest of the class to test the app. Students could interact with their own animated face via touch (sneezing, coughing, etc; see Multimedia Appendix 1). In front of their peers and teachers, they could display their image as a non-smoker/smoker 1, 3, 6, 9, 12, or 15 years in the future (see Figures 1-4). Multiple device displays can be projected simultaneously, which we used to consolidate the altering measures with graphics (eg, to explain wrinkle formation). We implemented mirroring with Galaxy Tab A (Samsung) via Apple’s proprietary AirPlay interface using the Android app “Mirroring360” (Splashtop Inc.).

In the second 10-minute phase, students were encouraged to try the app on their own device or one of the tablet computers provided for students who do not own a smartphone or did not download the app. The number of provided tablet computers was calculated so the phase would take up to 10 minutes at the most, factoring in a utilization time of about 2 minutes per student and a rate of 50% of students having downloaded the app. By this calculation, 20 minutes of the mirroring intervention and 10 provided tablets were sufficient to have every student within a grade of 100 pupils successfully photoaged at least once.

**Figure 5:**
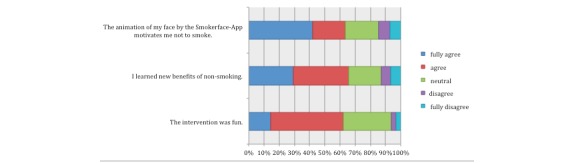
Main results of the survey.

The perception of the intervention by the students was measured directly after the intervention via three items in an anonymous survey (see Figure 5) asking (1) “The animation of my 3D-selfie motivates me not to smoke,” (2) “I learned new benefits of non-smoking,” and (3) “The intervention was fun.”

## Results

In our sample, we measured more than 60% (Item 1: 79/125; Item 2: 81/125; Item 3: 77/125) agreement on all three items (see Figure 5). Only a small fraction disagreed or fully disagreed that they learned new benefits of non-smoking (16/125, 12.8%) or that they were themselves motivated not to smoke (18/125, 14.4%). Nearly three-quarters (90/125, 72.0%) of students had already tried the app on their own or a friend’s device before the intervention, due to our letter.

The theoretical background of the participant-centered mirroring intervention includes increasing perceived self-efficacy of using the app, which has been proven to encourage repetitive use and is associated with the effectiveness of an intervention according to the theory of planned behavior [13]. Accordingly, 36% (45/124) fully agreed or agreed directly after the intervention that they wanted to use the app again on their own despite the one-time-use nature of the app and the fact that most of them had used the app at least twice already (neutral=32/124; disagree/fully disagree=47/124; missing=1). By causing direct peer group and teacher reactions to the intervention itself, the subjective norm is affected, which also predicts adolescent smoking [13].

The strongest predictor of adolescent smoking was perceived behavioral control (eg, if students think they could refuse a cigarette successfully) [13]. To this end, an age-appropriate reason not to smoke was integrated into the students’ community by means of the app name, “Smokerface”. After the intervention, many students would refer to smokers as “smokerfaces” or, when asked why they would not smoke, they stated that they did not want to be a “smokerface.” A majority (90%) of the students also agreed that the app was an appropriate tool to convince peers not to smoke when asked after the intervention. Smoking students motivated to quit were offered free science-based quit advice by the app.

## Discussion

### Principal Considerations

No evaluation of a tobacco-prevention program using photoaging is available to date despite the fact that mobile phone and tablet use among adolescents is constantly rising both in Germany and globally, providing a novel opportunity for health education [14]. Moreover, authors of dermatology papers as well as those of a recent Cochrane analysis have called for novel public health approaches concerning tobacco prevention [4,5].

The mobile phone app we investigated in this study was easy to implement and was well received. It can be added to existing school-based tobacco-prevention programs.

### Limitations

As this study was conducted only in Germany, our results might not be generalizable to other cultural or national settings. However, cosmetics are used by adolescents in the majority of countries and the World Health Organization is reportedly concerned about the tobacco industry increasingly targeting young females in their advertisements, especially in developing and emerging countries [1]. These developments increase the international relevance of our research.

### Conclusion

In summary, we present a novel way of integrating photoaging in school-based tobacco prevention, which affects the students’ peer group and considers the predictors of smoking in accordance with the theory of planned behavior. Further research is necessary to evaluate the intervention’s effects on the smoking prevalence of adolescents.

A consequence of this pilot study is an integration of the mirroring intervention into our curriculum, which will be evaluated in the Education Against Tobacco Randomized Trial in Germany with approximately 200 secondary schools, 15 medical schools, and around 400 medical students participating.

## Appendix

Multimedia Appendix 1 3D animation of a selfie via the Smokerface app illustrating one of the touch effects (cough) the students can trigger on their own selfies.
